# Impact of Chemotherapy in the Adjuvant Setting of Early Stage Uterine Leiomyosarcoma: A Systematic Review and Updated Meta-Analysis

**DOI:** 10.3390/cancers12071899

**Published:** 2020-07-14

**Authors:** Alessandro Rizzo, Margherita Nannini, Annalisa Astolfi, Valentina Indio, Pierandrea De Iaco, Anna Myriam Perrone, Antonio De Leo, Lorena Incorvaia, Valerio Di Scioscio, Maria Abbondanza Pantaleo

**Affiliations:** 1 Department of Experimental, Diagnostic and Specialty Medicine, S.Orsola-Malpighi Hospital, University of Bologna, 40128 Bologna, Italy; rizzo.alessandro179@gmail.com (A.R.); maria.pantaleo@unibo.it (M.A.P.); 2 Medical Oncology Unit, S.Orsola-Malpighi University Hospital, 40128 Bologna, Italy; 3 Department of Morphology, Surgery and Experimental Medicine, University of Ferrara, 44121 Ferrara, Italy; annalisa.astolfi@unife.it; 4 “Giorgio Prodi” Cancer Research Center, University of Bologna, 40128 Bologna, Italy; valentina.indio2@unibo.it; 5 Gynecologic Oncology Unit, S.Orsola-Malpighi University Hospital, 40128 Bologna, Italy; pierandrea.deiaco@unibo.it (P.D.I.); myriam.perrone@aosp.bo.it (A.M.P.); 6 Pathology Unit, S.Orsola-Malpighi University Hospital, 40128 Bologna, Italy; antonio.deleo@unibo.it; 7 Department of Surgical, Oncological and Oral Sciences, Section of Medical Oncology, Palermo University Hospital, 90100 Palermo, Italy; lorena.incorvaia@unipa.it; 8 Radiology Unit, S.Orsola-Malpighi University Hospital, 40128 Bologna, Italy; valerio.discioscio@aosp.bo.it

**Keywords:** meta-analysis, uterine leiomyosarcoma, uterine sarcoma, adjuvant therapy, chemotherapy

## Abstract

Background: Although the use of adjuvant chemotherapy (AC) appears to be increasing over the past few years, several clinical trials and previous meta-analyses failed to determine whether AC could improve clinical outcomes in uterine leiomyosarcoma (uLMS). The aim of this systematic review and meta-analysis was to compare AC (with or without radiotherapy) versus observation (obs) after primary surgery in early stage uLMS. Materials and Methods: Randomized controlled (RCTs) and non-randomized studies (NRSs) were retrieved. Outcomes of interest were as follows: distant recurrence rate, locoregional recurrence rate and overall recurrence rate. Results about distant recurrence rate, locoregional recurrence rate and overall recurrence rate were compared by calculating odds ratios (ORs) with 95% confidence intervals (CIs); ORs were combined with Mantel–Haenszel method. Results: Nine studies were included in the analysis, involving 545 patients (AC: 252, obs: 293). Compared with obs, AC did not reduce locoregional and distant recurrence rate, with a pooled OR of 1.36 and 0.63, respectively. Similarly, administration of AC did not decrease overall recurrence rate in comparison to obs. Conclusion: According to our results, AC (with or without radiotherapy) did not decrease recurrence rate in early stage uLMS; thus, the role of AC in this setting remains unclear.

## 1. Introduction

Uterine leiomyosarcoma (uLMS) is a rare tumor accounting for approximately 2% of all uterine malignancies and 65% of all uterine sarcomas, with an estimated incidence of 0.60 per 100.000 women/year [1,2,3,4,5]. Currently, uLMSs are staged in accordance with the Federation of Gynecology and Obstetrics (FIGO) 2009 staging system, which does not include tumor grading [6,7] (Table 1).

Total hysterectomy represents the therapeutic mainstay for localized disease [2,3] and, although there are no available data indicating that oophorectomy improves survival outcomes, bilateral salpingo–oophorectomy is considered a reasonable option in perimenopausal and postmenopausal women [8,9]. However, despite radical surgery, the risk of recurrence remains high, ranging between 50% and 70% [10] and to date, age, tumor stage and tumor size are recognized as prognostic factors [9,11,12] in resected uLMS. Additional prognostic factors are represented by tumor morcellation [13,14,15], extrauterine spread, mitotic index and tumor grade, although the prognostic value of tumor grade in uLMS remains controversial and it is not commonly applied to the staging procedures [3].

Despite the high recurrence rate of uLMS provides the rationale for postoperative treatment, neither adjuvant cytotoxic chemotherapy (AC) nor adjuvant radiotherapy (AR) have been shown to reduce the risk of relapse and to improve clinical outcomes in stage I–II uLMS [16]. In fact, studies on AC have reported controversial results and the clinical efficacy of adjuvant treatment including chemotherapy and/or radiation therapy is yet to be defined, most notably in stage I–II disease [17]. Although there is currently neither retrospective nor prospective evidence to support adjuvant treatment in uLMS, the last decade has seen an outstanding increase in the use of AC [18,19]. More specifically, a recent study by Littell et al. on 110 cases of early stage uLMS suggested that the proportion of patients receiving gemcitabine–docetaxel as AC increased from 6.5% in 2006–2008 to 46.9% of women between 2009–2013 [20]. Moreover, a survey by the Korean Gynecologic Oncology Group (KGOG) recently found that 42.3% of included physicians recommended AC and/or adjuvant radiation therapy after primary surgery in early stage uLMS [21].

To date, the attempt to translate in the adjuvant setting the use of agents with a high objective response rate in metastatic disease has not led to noteworthy results and this long-standing therapeutic “dilemma” has led to different hypothesis [22]. Several authors have suggested that the reasons could be the use of the wrong drugs or regimens, the wrong selection of patients and, lastly, the inclusion of an inadequate number of patients to find a difference between AC/AR and observation in available randomized controlled trials (RCTs) and non-randomized studies (NRSs) [23]. The decision whether to use AC in early stage uLMS currently represents a critical research question in medical oncology, something also witnessed by the number of recently published studies on this topic. Herein, we conducted a systematic review and updated meta-analysis to investigate the role of AC on the risk of recurrence after surgery in stage I–II uLMS patients.

## 2. Materials and Methods

### 2.1. Search Strategies

All clinical trials published from June 15, 1985 to March 29, 2020, which evaluated the effect of adjuvant chemotherapy in completely resected early stage uLMS were independently searched in PubMed/ Medline (https://www.medline.com/), Cochrane library (https://www.cochranelibrary.com/) and EMBASE databases (https://www.embase.com/) by two different authors (A.R. and M.N.). The following keywords were used: “adjuvant chemotherapy” OR “adjuvant treatment” OR “postoperative chemotherapy” AND “uterine sarcoma” OR “uterine leiomyosarcoma”; only articles written in English language and published in peer-reviewed journals were considered. Furthermore, proceedings of the main international oncological meetings (European Society of Medical Oncology, American Society of Clinical Oncology, American Association for Cancer Research, European Council of Clinical Oncology), were also searched from 1985 onward for relevant abstracts.

### 2.2. Selection Criteria

Studies selected from first analysis were then restricted to: (1) two-arm trials comparing AC versus observation; (2) studies with available data about recurrence rate.

### 2.3. Data Extraction and Quality Assessment

The following data were extracted for each publication: (1) study general information; (2) primary site; (3) interventions; (4) number of patients; (5) baseline characteristics of patients; (6) recurrence rate. Two independent authors (AR and MN) evaluated all studies, verifying the inclusion criteria.

The methodological quality of the NRSs were assessed using the nine-star Newcastle-Ottawa scale (NOS) [24]; based on standard quality assessment, studies with five or more stars were defined as high-quality studies. Conversely, the methodological quality of the RCTs were assessed using Cochrane collaboration tool, according to the grading of recommendations, assessment, development and evaluation (GRADE) guidelines [25]. This meta-analysis was conducted in accordance with preferred reporting items for systematic review and meta-analyses (PRISMA) guidelines [26].

### 2.4. Statistical Design

All statistical analyses were performed using R studio software. odds ratios (ORs) were used to analyze dichotomous variables, including distant recurrence rate, locoregional recurrence rate and overall recurrence rate; Mantel–Haenszel method was used in order to combine ORs. The chi-squared test and the I^2^ statistic examined the statistical heterogeneity between studies. We analyzed quantitative data using a fixed-effect model when I^2^ < 50% or a random-effect model in case of substantial heterogeneity.

### 2.5. Types of Outcome Measures

We examined 4 outcomes including distant recurrence rate (AC versus observation), locoregional recurrence rate (AC versus observation), overall recurrence rate (AC versus observation) and overall recurrence rate (AC ± radiotherapy versus observation).

## 3. Results

### 3.1. Studies Selected

**Figure 1:**
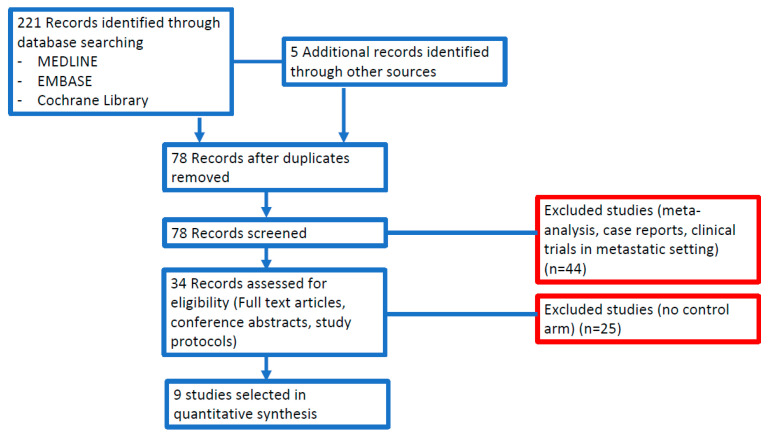
Study flow diagram.

Through the process of searching, we identified 226 potentially relevant reports, which were subsequently restricted to 9 after independent evaluation of two authors (AR and MN) [11,19,26,27,28,29,30,31,32]. We excluded 217 records as nonpertinent reports (meta-analysis and systematic reviews, review articles, editorials, case reports, preclinical studies, retrospective studies, non-randomized studies without the observation arm, single-arm studies, ongoing trials/trials in progress). Figure 1 reports PRISMA flow chart.

All studies included in our analysis were published as full text.

Of the 9 eligible studies, two [27,28] were RCTs, whereas the other seven were NRSs [11,20,29,30,31], all comparing adjuvant chemotherapy versus observation in completely resected, early stage (I–II) uLMS. Table 2. presents a summary of the included studies.

From these selected studies, a total of 545 patients were included (adjuvant treatment, chemotherapy with or without radiotherapy: 252; observation: 293). The chemotherapy regimens used in selected studies are described in Table 2. Since outcomes were not stratified according to type of chemotherapy, it was not possible to assess the efficacy of different regimens. All studies included in our analysis were judged as studies with high risk of bias, following independent evaluation by two authors (AR and MN).

### 3.2. ORs of Distant Recurrence Rate (Adjuvant Chemotherapy versus Observation)

**Figure 2:**
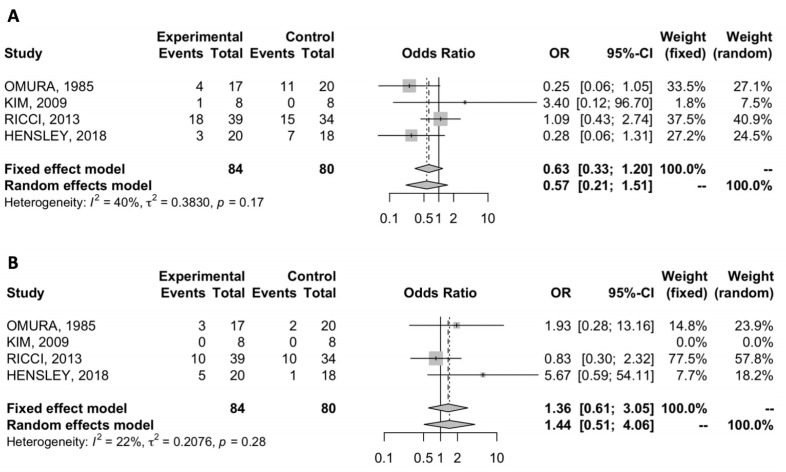
(**A**) Forest plot of comparison between adjuvant chemotherapy versus observation; the outcome was OR of distant recurrence rate; (**B**) forest plot of comparison between adjuvant chemotherapy versus observation; the outcome was OR of locoregional recurrence rate.

Four studies reported the distant recurrence rate of adjuvant chemotherapy and observation [27,28,29,30]. We compared distant recurrence rate in the two groups and no statistically significant differences were observed, with a pooled OR of 0.63 (95% CI = 0.33–1.20) (Figure 2A). The analysis showed low heterogeneity between trials (I^2^ = 40%), so a fixed-effects model was used.

### 3.3. ORs of Locoregional Recurrence Rate (Adjuvant Chemotherapy versus Observation)

Locoregional recurrence rate was reported in four studies [27,28,29,30]. Our analysis showed no statistically significant differences between adjuvant chemotherapy and observation, with a pooled OR of 1.36 (95% CI = 0.61–3.05) (Figure 2B). There was low heterogeneity present in the data (I^2^ = 22%).

### 3.4. ORs of Overall, Recurrence Rate (Adjuvant Chemotherapy versus Observation)

**Figure 3:**
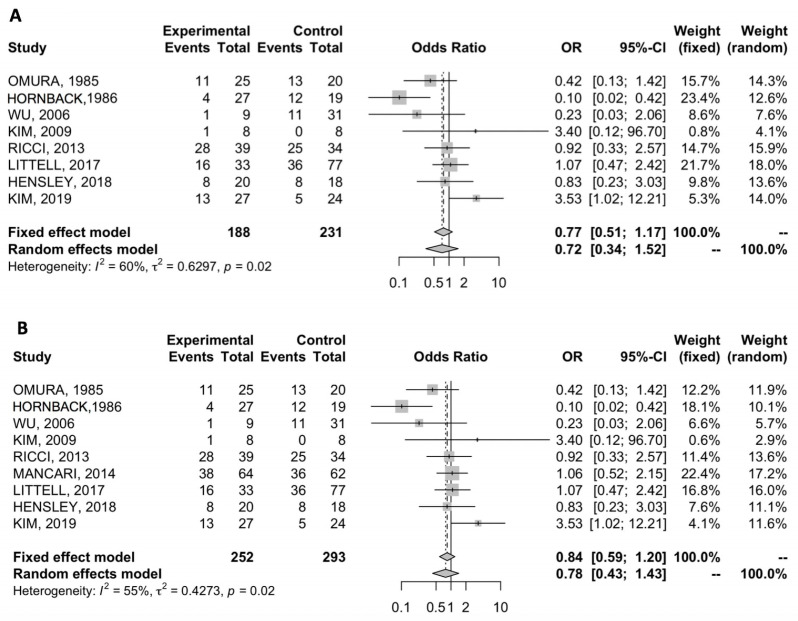
(**A**) Forest plot of comparison between adjuvant chemotherapy versus observation; the outcome was OR of overall recurrence rate; (**B**) forest plot of comparison between adjuvant chemotherapy versus observation; the outcome was OR of overall recurrence rate radiation therapy; the outcome was odds ratio of overall recurrence rate.

Eight studies included in our meta-analysis reported the overall recurrence rate in patients receiving adjuvant chemotherapy versus observation and no statistically significant differences were observed between the two groups, with a pooled OR of 0.72 (95% CI 0.33–1.52) (Figure 3A) [11,20,27,28,29,30,31,32,33]. The analysis detected a substantial level of heterogeneity (I^2^ value of 60%) and a random-effects model was adopted.

Similarly, all the nine studies reported the overall recurrence rate in patients treated with ac with or without radiotherapy versus observation [11,20,27,28,29,30,31,32,33]. We compared the overall recurrence rate in the two groups and no differences were detected (OR 0.78, 95% CI 0.43–1.43) (Figure 3B). A significant heterogeneity between trials (I^2^ = 55%) was detected, thus a random-effects model was used.

## 4. Discussion

Adjuvant treatment in early stage uLMS is a controversial and long-standing issue, given the conflicting results achieved in several RCTs and NRSs while assessing different agents in this setting [2]. Even though more aggressive surgical approaches have improved the chances of achieving radical resection, recurrence rates after primary surgery in uLMS remain high, ranging from 50% to 70% within first five years [34]. In our study, we conducted a meta-analysis of nine selected trials aimed at comparing the efficacy of AC versus observation in stage I–II uLMS. We found no statistically significant difference in distant recurrence rate, locoregional recurrence rate and overall recurrence rate between the two groups.

Regarding the overall recurrence rate, we performed two types of analyses: (1) AC versus observation (2) AC with or without radiotherapy versus observation. In both analyses, adjuvant treatment did not provide any significant benefit; moreover, radiation therapy was included in one of the four analyses because in some trials pelvic radiotherapy was allowed at the discretion of the investigators. This inevitably introduced an important source of bias that may have affected the real impact of AC on the overall recurrence rate. Our results are consistent with previous similar meta-analyses by Bogani in 2016 and Chae in 2019, both showing that AC does not reduce the recurrence rate in stage I–II uLMS patients [35,36]. In particular, the meta-analysis by Bogani et al. published in 2016, included six studies, for a total of 360 patients with early stage uLMS, of which 145 (40%), 53 (15%) and 155 (43%) underwent chemotherapy (with or without radiotherapy), radiotherapy and observation, respectively [35]. The authors found that chemotherapy (with or without radiotherapy) does not provide an improved outcome in comparison to observation (OR: 0.79; 95% CI: 0.48, 1.29) or radiotherapy (OR: 0.90; 95% CI: 0.42, 1.94). Loco-regional recurrence rate was found to be similar between patients undergoing chemotherapy (with or without radiotherapy) or observation alone (OR: 0.84; 95% CI: 0.44, 1.60). However, the analysis suggested that patients undergoing AC (with or without radiotherapy) experienced a trend towards a lower risk of developing distant recurrences (OR: 0.49; 95% CI: 0.24, 1.03), but a higher risk of developing loco–regional recurrences (OR: 3.45; 95% CI: 1.02, 11.73) in comparison to patients undergoing radiotherapy. More recently, the meta-analysis by Chae et al. drew the same conclusion: among 12 selected studies (three randomized trials and nine observational studies) AC did not decrease the risk of recurrence compared with observation (OR: 0.65; 95% CI: 0.37, 1.15) [36]. Of note, in subgroup analyses (study design, surgical staging, gemcitabine/docetaxel regimen, type of AR), neither AC nor AR significantly decreased the risk of recurrence.

In comparison with the two meta-analyses by Bogani and Chae, the present analysis adds a recent Korean study comparing AC versus observation in early stage uLMS. Moreover, we focused our analysis on AC, and, with the exception of overall recurrence rate, we did not include more analyses about the role of radiation therapy, since this analysis was out of the aim of the present study in this review. In the present meta-analysis, we selected nine studies (seven NRSs and two RCTs) for a total of 545 patients included. As regards the 7 NRSs included, the retrospective multi-institutional series by Ricci et al. of 108 patients with high grade stage I–II uLMS, who underwent primary surgery followed by observation, AR or AC postoperatively, was included [30]. According to this study, after a median follow-up of 41.8 months, recurrence rate was of 73.5%, 65.7% and 71.8%, respectively, with no statistical differences (*p* = 0.012). However, extrapelvic recurrences were higher in the AR (95.2%) than in the observation (60%) or AC (64.3%) cohorts (*p* = 0.012).

In a previous multicenter Italian experience regarding 140 women treated between 1976 and 2011, no statistically significant differences in terms of recurrence rate were detected between AC and observation after a follow-up of 63 months [32]. More recently, since tumor morcellation has been recognized as a negative prognostic factor, Kim et al. have evaluated the effectiveness of adjuvant treatment for morcellated, stage I uLMS in a multicenter setting [31]. After a median follow-up of 50.5 months on a total of 55 resected stage I uLMS patients, no differences in OS between AC and surgery only groups were found (5-year rate, 92.0% vs. 90.4%; *p* = 0.959). Likewise, no statistically significant difference in progression-free survival (PFS) was observed (3-year rate, 46.1% vs. 78.2%; *p* = 0.069). Regarding the only two RCTs available until now, no differences in recurrence rate, PFS and OS were observed in the first RCT published in 1985, on 156 patients with stage I-II uterine sarcomas, of which 48 uLMS randomly assigned to AC with adriamycin for six months or to no further treatment [27]. In this study, pelvic irradiation (external or intracavitary) was optional before randomization. More recently, an open-label, two-arm, randomized phase III trial of gemcitabine plus docetaxel, followed by doxorubicin versus observation, was conducted in patients with uterus-limited, high-grade LMS [28]. Unfortunately, the study was prematurely closed owing to slow accrual, thus leaving this long-standing research question unanswered. Among the reasons for the poor accrual, several authors suggested the important difference between an observation alone arm and intensive treatment with eight cycles of cytotoxic chemotherapy in patients who had undergone surgery a few weeks earlier [23].

In accordance with the previously published meta-analyses, AC does not seem to have an impact on the recurrence rate in early stage uLMS. However, some considerations are needed. The first limitation shared by all these meta-analyses is that results should be interpreted with caution, due to the substantial heterogeneity of the available studies. In particular, this great heterogeneity includes differences in ethnicity of participants, study design, lack of standardized imaging for recurrence detection, surgical procedures, follow-up, outcomes evaluation and type of adjuvant cytotoxic regimens adopted during the time. Moreover, within this heterogeneity, the small sample size may also affect the data interpretation, together with the inclusion of several retrospective studies. Another key point to consider is the lack of a direct comparison between each regimen used, which could correlate with different outcomes in uLMS. No data regarding overall survival were available and thus, our analysis was limited to the bare risk of recurrence using ORs and we could not perform a time-to-event analysis through hazard ratios. Lastly, our systematic review and meta-analysis were conducted using aggregated data instead of individual patient data, thus our findings should be interpreted with caution.

Therefore, to date, the choice of whether to give adjuvant chemotherapy in early stage uLMS still remains an oncological dilemma in everyday clinical practice. Indeed, despite neither AC, nor hormone blockade, nor radiation have proven to reduce the risk of relapse in uLMS, expert opinions suggest considering AC in selected cases. In particular, according to NCCN and ESMO–EURACAN guidelines, observation alone is feasible in completely resected early stage uLMS and remains the standard approach [37,38]. Nevertheless, adjuvant treatment in cases at higher recurrence risk (e.g., tumor spillage, tumor morcellation, high grade uLMS) may be considered after multidisciplinary discussion [37,38]. In this uncertain scenario, one of the most important challenges in the near future will probably be to better select patients to enroll in AC trials [39]. In fact, in the era of tailor-made oncology, progress in the management of uLMS cannot prescind from a close collaboration between molecular biology and clinical oncology, orienting future researches towards the identification of the patients that could really benefit from AC [40,41].

## 5. Conclusions

According to our analyses, AC did not seem to reduce locoregional recurrence, distant recurrence and overall recurrence in early stage uLMS. Given the substantial heterogeneity affecting our analyses, our results should be interpreted with caution. To date, although AC is commonly administered in everyday clinical practice, its role in early stage uLMS after primary surgery has not yet been proven beneficial at all.

## Figures and Tables

**Table 1:** Federation of Gynecology and Obstetrics (FIGO) staging for uterine sarcomas.

FIGO Staging System for Uterine Sarcomas	
Stage	Definition
I	Tumor limited to uterus
IA	Less than 5 cm
IB	More than 5 cm
II	Tumor extends beyond the uterus, within the pelvis
IIA	Adnexal involvement
IIB	Involvement of other pelvic tissues
III	Tumor invades abdominal tissues
IIIA	One site
IIIB	More than one site
IIIC	Metastasis to pelvic and/or para-aortic lymph nodes
IV	
IVA	Tumor invades bladder and/or rectum
IVB	Distant metastasis

**Table 2:** Summary of the included studies.

Author/Year	Country	Study Design	Type of AC	Number of Patients Receiving Adjuvant Treatment	Number of Patients Receiving Observation	Included Stage	Outcomes Included in the Analysis
Omura et al. (1985) [27]	USA	RCT	adriamycin	AC: 17 AR: 11	20	I II	- Distant recurrence rate - Locoregional recurrence rate - Overall recurrence rate (AC vs. obs) - Overall recurrence rate (AC ± AR vs. obs)
Hornback et al. (1986) [33]	USA	NRS	doxorubicin	AC: 27 AR: 11	19	I II	- Overall recurrence rate (AC vs. obs) - Overall recurrence rate (AC ± AR vs. obs)
Wu et al. (2006) [11]	Taiwan	NRS	cisplatin/ifosfamide: 4 cisplatin/adriamycin/epirubicin: 4 cisplatin/adriamycin alternating with cisplatin/ifosfamide: 3 liposomal doxorubicin: 2 cisplatin/adriamycin/cyclophosphamide: 2 vincristine/adriamycin/cyclophosphamide: 1	AC: 9 AR: 1	31	I	- Overall recurrence rate (AC vs. obs) - Overall recurrence rate (AC ± AR vs. obs)
Kim et al. (2009) [29]	Korea	NRS	cisplatin/adriamycin cisplatin/adriamycin/ifosfamide	AC: 8 AR: 7	8	I	- Distant recurrence rate - Locoregional recurrence rate - Overall recurrence rate (AC vs. obs) - Overall recurrence rate (AC ± AR vs. obs)
Ricci et al. (2013) [30]	USA	NRS	gemcitabine/docetaxel: 23 doxorubicin/Cisplatin: 9 ifosfamide or doxorubicin: 5 topotecan: 2 cisplatin/ifosfamide: 1	AC: 39 AR: 35	34	I II	- Distant recurrence rate - Locoregional recurrence rate - Overall recurrence rate (AC vs. obs) - Overall, recurrence rate (AC ± AR vs. obs)
Mancari et al. (2014) [32]	Italy	NRS	doxorubicin/ifosfamide: 54 gemcitabine/docetaxel: 4 doxorubicin/dacarbazine: 2	AC: 64 AR: 14	62	I II	- Overall recurrence rate (AC ± AR vs. obs)
Littell et al. (2017) [20]	USA	NRS	gemcitabine/docetaxel: 31 gemcitabine/docetaxel/doxorubicin: 2	AC: 33	77	I	- Overall, recurrence rate (AC vs. obs) - Overall recurrence rate (AC ± AR vs. obs)
Hensley et al. (2018) [28]	USA	RCT	gemcitabine/docetaxel/doxorubicin: 20	AC: 20	18	II	- Distant recurrence rate - Locoregional recurrence rate - Overall recurrence rate (AC vs. obs) - Overall, recurrence rate (AC ± AR vs. obs)
Kim et al. (2019) [31]	Korea	NRS	docetaxel–gemcitabine: 8 doxorubicin–cisplatin: 4 doxorubicin–ifosfamide: 2 paclitaxel–carboplatin: 2 ifosfamide–paclitaxel: 2 ifosfamide–cisplatin: 1 etoposide–ifosfamide–cisplatin: 1 cyclophosphamide–vincristine–doxorubicin–dacarbazine: 1	AC: 21 ACR: 6 AR: 4	24	I	- Overall, recurrence rate (AC vs. obs) - Overall, recurrence rate (AC ± AR vs. obs)

AC: adjuvant chemotherapy. AR: adjuvant radiotherapy. ACR: adjuvant chemoradiotherapy. USA: United States of America. RCT: randomized controlled trials. NRS: non randomized studies.
